# P131: Cost-effectiveness of a team and leaders-directed strategy to improve nurses’ adherence to hand hygiene

**DOI:** 10.1186/2047-2994-2-S1-P131

**Published:** 2013-06-20

**Authors:** A Huis, M Hulscher, E Adang, R Grol, T van Achterberg, L Schoonhoven

**Affiliations:** 1 IQ Healthcare, Radboud University Nijmegen Medical Centre, Nijmegen, the Netherlands; 2 Epidemiology, Biostatistics and HTA, Radboud University Nijmegen Medical Centre, Nijmegen, the Netherlands; 3 Faculty of Health Sciences, University of Southampton, Southampton, UK

## Introduction

Many strategies have been designed and evaluated to address poor hand hygiene (HH) compliance. Unfortunately, well-designed economic evaluations of HH improvement strategies are lacking.

## Objectives

We compared the cost-effectiveness of two successful implementation strategies for improving nurses’ hand hygiene (HH) compliance and reducing hospital acquired infections (HAI’s).

## Methods

A cost-effectiveness analysis alongside a cluster randomised controlled trial was conducted in 67 nursing wards of three hospitals in the Netherlands, with inpatient wards as the unit of randomisation. The evaluation used a hospital perspective. The control group received a state-of-the-art strategy (SAS) including education, reminders, feedback and optimising materials and facilities. The experimental group received a team and leaders-directed strategy (TDS) which included all elements of the SAS supplemented with interventions aimed at social influence within teams and enhancing leadership. The most efficient strategy was determined by the incremental cost-effectiveness ratio per extra percentage of HH compliance gained, and the incremental cost-effectiveness ratio per additional percentage reduction in the HAI rate. Bootstrap methods were used to determine confidence intervals for these incremental cost-effectiveness ratio’s.Two scenarios of 15 and 30% were used to express the association between increased HH compliance and the reduction in HAIs

## Results

The TDS was significantly more effective in improving HH compliance. The mean difference effect was 8.91%. This extra increase was achieved at an average cost of € 5497 per ward. The incremental cost per extra percentage of HH gained on ward level was € 622. The incremental cost per additional percentage reduction in the HAI rate on ward level was € 2074 (30% scenario) and € 4125 (15% scenario). Within the 30% scenario, there is a probability of 90% that the TDS is cost-effective and within the 15% scenario, there is a probability of 70% that the TDS is cost-effective.

## Conclusion

Optimising hand hygiene compliance through a team and leaders-directed strategy is cost-effective as compared to a state-of-the-art strategy.

## Disclosure of interest

None declared

